# *RiboPlotR*: a visualization tool for periodic Ribo-seq reads

**DOI:** 10.1186/s13007-021-00824-4

**Published:** 2021-12-07

**Authors:** Hsin-Yen Larry Wu, Polly Yingshan Hsu

**Affiliations:** Department of Biochemistry & Molecular Biology, Michigan State University, East Lansing, MI 48824 USA

**Keywords:** Ribo-seq, 3-nucleotide periodicity, Isoform, Upstream ORF, Small ORF

## Abstract

**Background:**

Ribo-seq has revolutionized the study of genome-wide mRNA translation. High-quality Ribo-seq data display strong 3-nucleotide (nt) periodicity, which corresponds to translating ribosomes deciphering three nts at a time. While 3-nt periodicity has been widely used to study novel translation events such as upstream ORFs in 5′ untranslated regions and small ORFs in presumed non-coding RNAs, tools that allow the visualization of these events remain underdeveloped.

**Results:**

*RiboPlotR* is a visualization package written in R that presents both RNA-seq coverage and Ribo-seq reads in genomic coordinates for all annotated transcript isoforms of a gene. Specifically, for individual isoform models, *RiboPlotR* plots Ribo-seq data in the context of gene structures, including 5′ and 3′ untranslated regions and introns, and it presents the reads for all three reading frames in three different colors. The inclusion of gene structures and color-coding the reading frames facilitate observing new translation events and identifying potential regulatory mechanisms.

**Conclusions:**

*RiboPlotR* is freely available (https://github.com/hsinyenwu/RiboPlotR and https://sourceforge.net/projects/riboplotr/) and allows the visualization of translated features identified in Ribo-seq data.

## Background

**Fig. 1:**
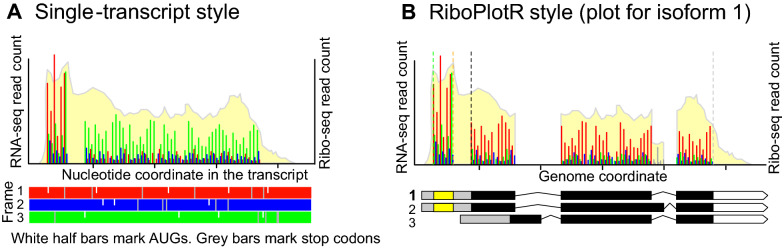
The commonly used single-transcript style versus the *RiboPlotR* style for a hypothetical gene**. A** Example of a commonly used single-transcript plot. RNA-seq and Ribo-seq reads are shown for one mature transcript isoform per plot. For Ribo-seq reads, either the most 5′ site or the P-site position (the first nt within the peptidyl site within the ribosome) is used for plotting. The first nt of the transcript is considered frame 1. Reads that are mapped to each reading frame are shown in red, blue, and green. Thus, the Ribo-seq reads for the annotated CDS can be in one of the three colors. AUG start codons are marked as white half-lines in all three frames. The stop codons are marked as grey lines in all three frames. **B** The *RiboPlotR* style for the same gene in **A** shows all annotated transcript isoform models in parallel with RNA-seq coverage and Ribo-seq P-site reads. Within the gene model, the grey boxes indicate 5′ UTRs, the black boxes indicate the annotated ORFs, and the white pentagonal arrows indicate 3′ UTRs. The isoform being considered is labeled in bold. In addition to the annotated ORF, one upstream ORF (yellow box in the gene models) can be shown in the same plot. For all transcript isoforms of a given gene, the same RNA-seq coverage and Ribo-seq P-sites are used for plotting. For the annotated ORF, the expected CDS range is marked between a black dashed line (translation start site) and a grey dashed line (translation stop site); for the uORF, the CDS range is marked between a green dashed line (translation start site) and an orange dashed line (translation stop site). The Ribo-seq P-sites that map to the expected frame, + 1 frame, and + 2 frame are marked in red, blue, and green, respectively. The Ribo-seq P-sites mapped outside of the expected CDS in either the annotated ORF or uORF are shown in grey. Thus, the majority of the Ribo-seq reads for the annotated CDS will be in red if the data agree with the annotation (here the isoform 1 is being considered). From the data, we can infer isoforms 1 and 2 are both expressed and translated, but isoform 3 is not expressed. Since we selected isoform 1 for plotting here, Ribo-seq P-sites that are unique to isoform 2 and not included in isoform 1 are marked in grey

mRNA translation is the last step in the central dogma of molecular biology. Despite its importance in directly controlling protein production, translation is much less well understood than transcription. In the past decade, ribosome profiling, also known as Ribo-seq, has revolutionized the study of translation by enabling the mapping and quantification of ribosome footprints on individual transcripts genome-wide [6]. High-quality Ribo-seq data display strong 3-nucleotide (nt) periodicity, which corresponds to ribosomes decoding 3 nts per codon. While many computational tools use 3-nt periodicity to facilitate the identification of novel translation events in the transcriptome [1, 2, 7, 19], visualizing the periodicity of Ribo-seq reads remains difficult. In most of the literature to date, the plots used for visualization either completely exclude periodicity [12, 14] or only focus on periodicity in the context of one mature transcript isoform [1, 8]. In the latter case, the Ribo-seq reads for each of the three reading frames are shown (Fig. 1A). This “single-transcript” style of plotting is useful for identifying overlapping translation events within a specific mature isoform but would miss Ribo-seq reads that belong to other isoforms, as well as those mapped to introns and unannotated coding exons.

Here, we describe the *RiboPlotR* package (Fig. 1B) for visualizing RNA-seq/Ribo-seq reads in the context of gene structures, including Ribo-seq reads mapped to the annotated coding sequences (CDSs), 5′ and 3′ untranslated regions (UTRs), and introns, with all annotated transcript isoform models displayed in parallel in the plot. There are several advantages to the style used in *RiboPlotR*: (1) we can detect novel translation events in the unannotated coding regions, such as those in the UTRs and introns; (2) by including all transcript isoform models in the plot, in most cases, we can visually determine which transcript isoform(s) is/are translated; (3) by comparing sequencing data and annotated gene models in parallel, we can identify discrepancies between the Ribo-seq data and the predicted CDSs, such as frameshifts and variations in coding regions; similarly, any discrepancies between the mRNA profile and the annotated transcript isoforms are also easily visualized; and (4) the relative Ribo-seq abundance in different transcript features, such as upstream ORFs (uORFs) and introns, can be visualized and thus used to infer potential regulatory mechanisms and generate new hypotheses. Below, we describe usages and examples with *RiboPlotR* to visualize translation events in a gene with a predicted uORF and in another gene with different transcript isoforms.

## Implementation

*RiboPlotR* uses base R commands for plotting. It requires the *GenomicRanges*, *GenomicFeatures*, *GenomicAlignments*, *Rsamtools* and *rtracklayer* packages from Bioconductor [9, 10, 15]. *RiboPlotR* reads in the transcriptome annotation files (gtf or gff3) with the *gene.structure* function, which is based on the *makeTxDbFromGFF* function from *GenomicFeatures*. The *gene.structure* function further processes the transcriptome annotation to retrieve the genomic coordinates of genes, transcripts, exons, CDSs, and UTRs. To visualize the uORFs, another gtf/gff3 file containing uORF coordinates can be loaded with the *uorf.structure* function. In addition, we implemented two methods to help users generate gtf files for uORFs (code available: https://github.com/hsinyenwu/RiboPlotR_addition). In the current version of *RiboPlotR*, users can visualize one uORF at a time. The *rna_bam.ribo* function reads in the RNA-seq-mapped bam file(s) and the Ribo-seq P-site coordinate file(s). The Ribo-seq P-site is denoted as the first nt of the peptidyl site within the ribosome. The RNA-seq bam files need to be mapped and sorted based on genomic coordinates. A *bai* (i.e., bam index) file is also required. We recommend users map the RNA-seq reads with the *STAR* aligner and sort/index the bam file with *samtools* [3, 11].

The Ribo-seq P-site coordinate file should be a tab-delimited file. From left to right, the Ribo-seq P-site coordinate file should contain “count”, “chromosome number”, “coordinate” and “strand” information, but without headers. For each chromosome coordinate, the column “count” should be the sum of the Ribo-seq P-site counts. The P-site information can be acquired from the *RiboTaper* output file “P_sites_all” or any other package that defines P-site positions. If the P-site information is obtained from *RiboTaper*, the user can use the Linux command “cut -f 1,3,6 P_sites_all | sort | uniq -c | sed -r 's/^( *[^]+) +/\1\t/' > name_output_file” to produce the P-site file.

*RiboPlotR* requires the following input files: (1) a gtf or gff3 file for transcriptome annotation, which should be recognizable with the *GenomicFeatures* package and the transcript name should be composed of a gene name, a period and a number (a widely used format for plant genome annotations); (2) mapped and coordinate-sorted bam file(s) for RNA-seq; and (3) tab-delimited file(s) for Ribo-seq P-site coordinates and counts. A gtf or gff3 file for uORF coordinates is optional. Moreover, users can read in one or two sets of bam and P-site files to compare translational profiles under two different conditions.

## Results and discussion

### Basic utility

*RiboPlotR* can be used to plot data from different organisms because it uses base R functions to input Ribo-seq P-site coordinates and uses widely adopted Bioconductor packages to input standard gtf/gff3 files and RNA-seq bam files. The *RiboPlotR* package contains a sample Ribo-seq dataset from Arabidopsis shoots and roots that was originally described in [5]. The sample RNA-seq data used here are a paired-end 100-bp dataset from the same study.

The basic workflow of *RiboPlotR* is:

1. Load the transcriptome annotation gtf/gff3 file containing the gene, transcript, exon, CDS and UTR ranges using *gene.structure*.
2. (Optional) To plot a uORF for a transcript, load the uORF gtf/gff3 file using the *uorf.structure* function.
3. Load the mapped and coordinate-sorted RNA-seq bam file and the Ribo-seq P-site position/count file by running *rna_bam.ribo*.
4. Use one of the four plot functions below and enter the gene name and isoform number to plot the translation of the isoform.

Four plot styles are available:

*PLOTc*: plots RNA-seq and Ribo-seq in one panel (plot **c**ompact).

*PLOTt*: plots RNA-seq and Ribo-seq separately in two panels (plot **t**wo).

*PLOTc2*: plots RNA-seq and Ribo-seq in one panel for two conditions.

*PLOTt2*: plots RNA-seq and Ribo-seq separately in two panels for two conditions.

### Plot presentations

*RiboPlotR* separately plots each transcript isoform of a given gene. Only one isoform is plotted at a time, and the default is to plot isoform 1. For each isoform, the same RNA-seq and Ribo-seq reads are used for plotting; the only difference is the expected coding region for the Ribo-seq reads, which is defined by a black dashed line (expected translation start) and a grey dashed line (expected translation stop). Inside the expected coding region, Ribo-seq P-sites that are mapped in the expected frame, the + 1 frame, and the + 2 frame are presented using red, blue and green lines, respectively. Ribo-seq P-sites that are outside the expected coding region are shown in grey. Thus, for high-quality datasets, most of the P-sites will be in red if the data agree with the annotation. The x-axis below the gene models indicates the genomic coordinates, whereas the primary y-axis (left) indicates the RNA-seq count, and the secondary y-axis (right) indicates the Ribo-seq P-site count. When an isoform is translated, the majority of P-sites should cover the expected CDSs and are shown in red. If two isoforms cover different coding frames at the 3′ ends, the two plots will have different color schemes at the 3′ end. This design allows users to quickly see if a plotted isoform is being actively translated (see examples below). Below, we provide two examples of how to interpret *RiboPlotR* plots. The code for examples below is available on github (https://github.com/hsinyenwu/RiboPlotR).

### Plotting without or with a uORF

**Fig. 2:**
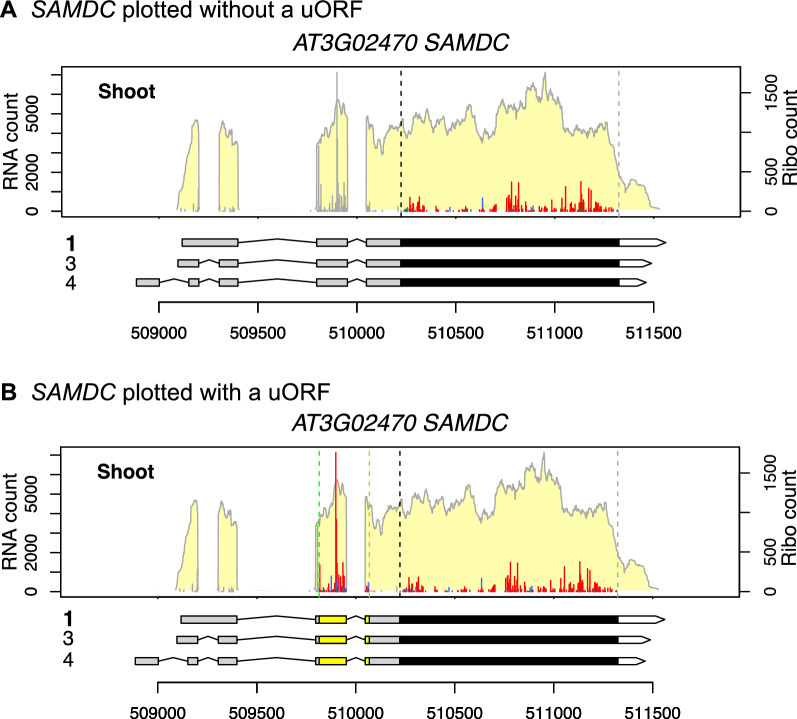
Plotting without or with a uORF. The *AT3G02470* gene is plotted using the PLOTc function of *RiboPlotR*. **A** Only the annotated ORF is considered. The Ribo-seq P-site reads outside of the annotated CDS are marked in grey. **B** Both the annotated ORF and CPuORF9 are considered. The data are presented as described in the Fig. 1 legend; genomic coordinates are shown below the gene models

Figure 2 shows *AT3G02470*, which encodes S-Adenosylmethionine decarboxylase (SAMDC), plotted using the *PLOTc* function. In addition to the abundant Ribo-seq reads in the expected CDS, many reads are present in the 5′ UTR (Fig. 2A). The Ribo-seq reads in the 5′ UTR imply two possibilities: the 5′ UTR reads could be from a uORF(s) or result from the usage of a non-AUG translation start site. *SAMDC* is known to have a conserved peptide uORF (CPuORF9) [4], which is annotated as *AT3G02468* in TAIR10. We plotted *AT3G02470* again with the 'uORF = "AT3G02468"' option. The default of uORF isoform is 1 and can be changed using the “uORFisoform” parameter. The Ribo-seq reads in the 5′ UTR are clearly highly enriched in the predicted CDS for CPuORF9 and are consistent with the expected reading frame (Fig. 2B). Thus, CPuORF9 is highly translated in the condition examined here. This example demonstrates that *RiboPlotR* is useful for visualizing additional translation events outside of the annotated CDS within a gene.

Some transcript isoforms have identical CDSs, and their only differences are in the UTRs. In these cases, we can use the RNA-seq plot to determine which isoform(s) is/are expressed. For example, the three isoforms of *SAMDC* share the same CDS (Fig. 2). However, based on the RNA-seq coverage and the exon–intron structures, it is clear that only isoform 3 is expressed.

### Plotting different transcript isoforms

**Fig. 3:**
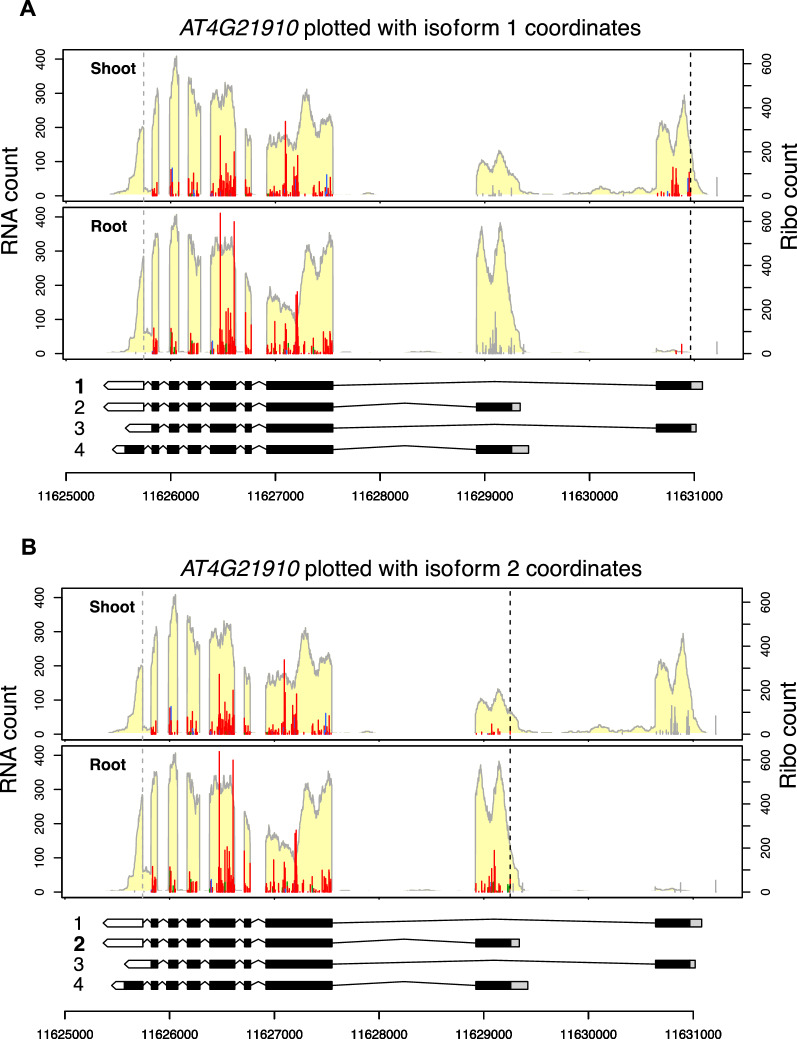

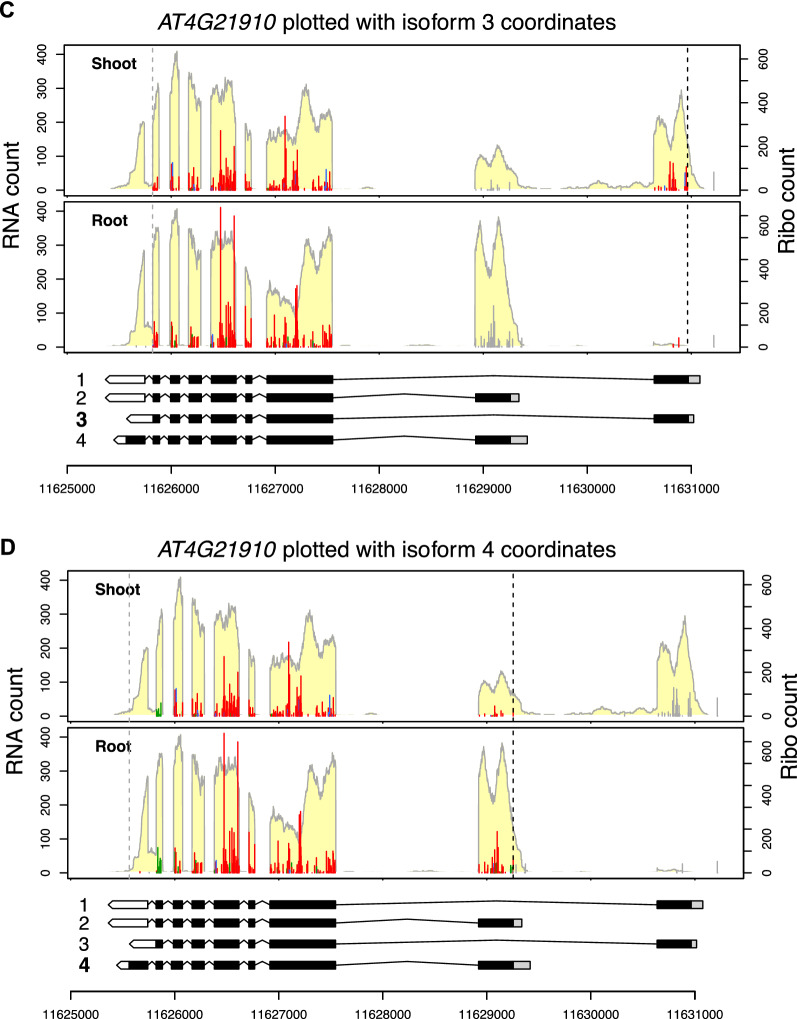
Plotting different transcript isoforms. The *AT4G21910* gene is plotted using the *RiboPlotR* PLOTc2 function to compare the data in Arabidopsis shoot and root. Transcript isoforms 1 to 4 of the *AT4G21910* gene are plotted in **A** to **D**, respectively. Data are presented as described in the Fig. 1 legend; genomic coordinates are shown below the gene models

Figure 3 shows *AT4G21910*, which encodes a multidrug and toxic compound extrusion (MATE) efflux family transporter, plotted using the *PLOTc2* function and comparing data from the shoot and root. In this case, the first two isoforms are the major translated isoforms since the RNA-seq/Ribo-seq coverage and frame color are consistent with these two isoforms (Fig. 3A and B). Moreover, isoform 1 is preferentially transcribed and translated in the shoot, while isoform 2 is preferentially transcribed and translated in the root (Fig. 3A and B). Based on the RNA-seq coverage of the first and last exons, isoform 3 is not significantly expressed in the shoot or root. Rather, an unannotated isoform, which is similar to isoform 2, has the last intron retained in the root (Fig. 3C). Isoform 4 is not considerably expressed or translated, either (Fig. 3D); there are very few Ribo-seq reads in the last exon of isoform 4, and the Ribo-seq reads in the second-to-last exon are not in the expected reading frame (instead of being red, they are green). This result indicates that the predicted coding region of isoform 4 is not used. In other words, if isoform 4 were actively translated, the Ribo-seq reads in the second-to-last exon would be in red, and there would be many reads in the CDS within the last exon of isoform 4. Finally, the RNA-seq coverage indicates that the shoot sample expresses an isoform with the first intron retained (Fig. 3A), suggesting that intron retention or another undefined isoform exists in the shoot. A cautious note is that the full mRNA structure of the transcripts cannot be determined by short-read sequencing and might be missing in annotations. Long-read sequencing or other methods may be required to identify the actual structure of unknown transcripts and help to interpret the Ribo-seq data.

### Other considerations

The reads plotted for the two conditions (i.e., shoot and root) are not normalized. If gene expression under the two conditions being studied is comparable, users can normalize the reads by randomly selecting a fixed total amount of reads from the RNA-seq bam file and Ribo-seq P-sites between the two conditions. However, normalization to the same RNA-seq and Ribo-seq read counts could be easily skewed by changes in the expression of highly expressed genes. For differential expression/translation analyses, users should use other software packages designed for those purposes (e.g., *DESeq2* or *Xtail*) [13, 18]. The *RiboPlotR* package is primarily for visualizing read distribution.

Ribo-seq is particularly useful for discovering novel translational events, such as uORFs in 5′ UTRs and small ORFs translated within presumed non-coding RNAs. Since these novel translation events are not part of formal annotations, users must generate a customized gtf/gff3 file to include the coordinates of these novel ORFs for data visualization in *RiboPlotR*. Similarly, if users wish to visualize the usage of non-AUG start sites, they can modify the CDS ranges of their genes of interest in the input annotation file. More examples of using *RiboPlotR* to explore unannotated translation events can be found in our recent publication on the Arabidopsis and tomato translatomes [5, 16, 17].

## Conclusions

In conclusion, *RiboPlotR* combines a transcriptome annotation file, a standard RNA-seq bam file, and a Ribo-seq P-site position/count file to plot the RNA-seq coverage and Ribo-seq P-site positions with genomic coordinates for each isoform considered. It can be used to investigate the translation of specific transcript isoforms, uORFs, and other novel translational events. This software has been tested in Arabidopsis and tomato, and we welcome users’ feedback to help us improve and update the software.

### Availability and requirements

Project name: *RiboPlotR*.

Project home pages: https://github.com/hsinyenwu/RiboPlotR and https://sourceforge.net/projects/riboplotr/.

Operating system(s): Platform independent.

Programming language: R.

Other requirements: R Bioconductor packages: *GenomicRanges*, *GenomicFeatures*, *GenomicAlignments*, *Rsamtools* and *rtracklayer.*

License: GPL-3.

Restrictions on use by non-academics: None.

## Data Availability

Not applicable.

## Abbreviations

ORF: Open reading frame
nt: Nucleotide
CDS: Coding sequence
UTR: Untranslated region
uORF: Upstream ORF
